# Heavy rainfall triggers increased nocturnal flight in desert populations of the Pacific black duck (*Anas superciliosa*)

**DOI:** 10.1038/s41598-017-17859-0

**Published:** 2017-12-14

**Authors:** J. F. McEvoy, R. F. H. Ribot, J. C. Wingfield, A. T. D. Bennett

**Affiliations:** 1 Smithsonian Conservation Biology Institute, 1500 Remount Road, Front Royal, VA 22630 USA; 2 Centre for Integrative Ecology, Deakin University, Locked Bag 20000, Geelong, VIC 3220 Australia; 3 Department of Neurobiology, Physiology and Behaviour, University of California One Shields Avenue, Davis, California 95616 USA

## Abstract

Understanding of avian nocturnal flight comes mainly from northern hemisphere species in seasonal temperate ecosystems where nocturnal flight is often precisely timed and entrained by annual photoperiod. Here we investigate patterns of nocturnal flight in waterbirds of Australian desert ecosystems that fly considerable distances to find temporary water bodies formed from rainfall which is highly unpredictable seasonally and spatially, and when there is sufficient water, they then breed. How they perform these feats of navigation and physiology remain poorly known. Using GPS tracking of 38 satellite tagged Pacific black ducks (*Anas superciliosa*) in two contrasting ecosystems, before and after heavy rainfall we revealed a key role for facultative nocturnal flight in the movement ecology of this species. After large rainfall events, birds rapidly increased nocturnal flight activity in the arid aseasonal ecosystem, but not in the mesic seasonal one. Nocturnal flights occurred throughout the night in both ecosystems. Long range flights (>50 km in 2 hours) occurred almost exclusively at night; at night the distance flown was higher than during the day, birds visited more locations, and the locations were more widely dispersed. Our work reveals that heavy rainfall triggers increased nocturnal flight activity in desert populations of waterbirds.

## Introduction

Levels of nocturnal activity in animals change over time in response to environmental cues such as lunar cycles^1–4^, annual photoperiod, temperature, rainfall and food availability^5–7^. In some migratory bird species, annual changes in day length trigger changes in physiology and behaviour in preparation for migration at night, via an endogenous ‘clock’ that regulates the rhythm of diurnal and nocturnal activity^8–12^. These normally diurnal birds commence nocturnal migratory restlessness on days coinciding with their migratory departures^13–15^. Outside of this well-studied model system, however, surprisingly little is known about the nocturnal life of bird species presumed to be diurnal. Some taxa are known to be active during the day and night, particularly waterfowl^16–19^. They switch habitats at dawn and dusk^20,21^, but little is known about movement between these times, nor the potential role of facultative nocturnality as an adaptation for responding to rapid changes in the environment^22,23^.

Improved understanding of avian nocturnal flight has benefits spanning from conservation, such as better estimates of population size and reserve design^24^, to potentially human health, as long-distance flights of waterfowl have often been implicated in the global spread of avian influenza^25^. Additionally, ENSO (El Niño–Southern Oscillation) events cause extended drought and floods for about half the world’s human population, including most countries in or bordering the Pacific and Africa’s East coast; ENSO has also been implicated in causing human influenza pandemics, with the hypothesized mechanism being changes in waterfowl movements^26^. To date, ENSO has been linked to changes in the movement^27,28^, population density^29^ and moulting schedule^30^ of some migratory bird species, including passerines, raptors and waterfowl, but tests of the effects of ENSO on nocturnal flight in waterfowl (or any bird species) have yet to be conducted. Our work aims to fill this major gap in knowledge.

**Figure 1:**
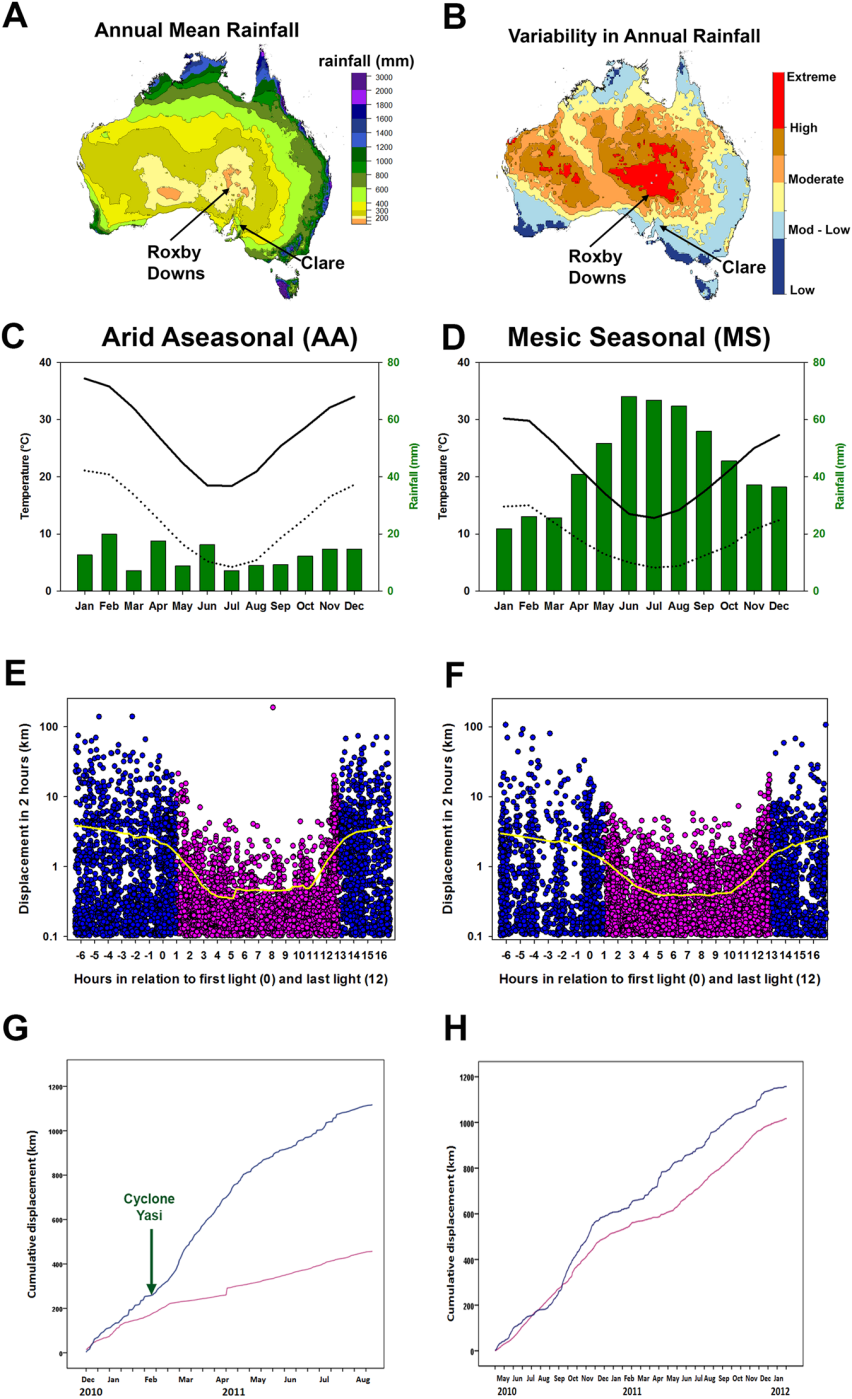
Climate data for two ecosystems; arid aseasonal (Roxby Downs AA, left column); and mesic seasonal (Clare MS, right column). Annual mean rainfall (**A**,**C**,**D**) variability in annual rainfall (**B**) and temperature (**C**,**D**) and diurnal (pink) and nocturnal (blue) movement (**E** and **F**; yellow lines produced using a negative exponential smoothing technique with a sampling proportion of 0.2) and cumulative displacement, between consecutive GPS fixes at 2 hour intervals (**G** and **H**), of tagged Pacific black duck. The x-axis in (**E**) and (**F**) represents time in hours relative to first light (indicated by 0 hours) and last light (indicated by 12 hours) at the location of each tagged bird; thus negative hours indicate values before first light. Annual mean rainfall data was averaged over at least 18 years. Variability in rainfall was calculated as the 90^th^ rainfall percentile minus the 10^th^ rainfall percentile divided by the 50^th^ percentile and temperature (averaged over at least 18 years) sourced from Australian Bureau of Meteorology (sources: http://www.bom.gov.au/jsp/ncc/climate_averages/rainfall/index.jsp, http://www.bom.gov.au/jsp/ncc/climate_averages/rainfall-variability/index.jsp, made available under a creative commons 3.0 licence; https://creativecommons.org/licenses/by/3.0/au/; labels have been added to indicate trapping sites).

Australia’s inland deserts are characterized by low mean annual rainfall (Fig. 1A), which is unpredictable in time and space (Fig. 1B,C). The temperate coastal mesic ecosystems, by contrast, have predictable rainfall and temperature regimes (Fig. 1D). In Australia El Niño is associated with drought and La Niña with flooding. Rain events, such as those caused by tropical cyclones associated with La Niña, can have a transformative effect on inland desert ecosystems^31,32^. For waterfowl, deserts can change overnight from a few permanent water bodies separated by a terrestrial matrix, to a vast network of resource rich, yet temporary, wetland habitat^33^. Successful reproduction for many species depends on an ability to respond rapidly and effectively to such massive changes in resource availability^32,34–38^. Under such conditions, many nomadic waterbirds move inland from coastal refugia^39,40^, and some species can remain inland throughout long drought periods^41^. During these ‘boom’ periods waterfowl may breed continuously until conditions deteriorate^33,42,43^. How nomadic waterbirds know where and when it has rained inland, is an enduring mystery. So too are the physiological and behavioural adaptations that allow waterbirds to successfully exploit these ecosystems.

We used two-hourly GPS satellite tracking of 38 Pacific black ducks (*Anas superciliosa*) to compare flights during the day and night before and after large rainfall events in contrasting ecosystems, one Arid Aseasonal (AA) and the other Mesic Seasonal (MS), matched for longitude. If exploratory flights are part of the behavioural adaptations allowing exploitation of ephemeral desert wetlands, we expected that major rainfall events would trigger increased flight in the AA ecosystem but not in the MS ecosystem.

## Results

### Nocturnal flight distances

In both ecosystems, Arid Aseasonal (AA) and Mesic Seasonal (MS), Pacific black ducks moved greater distances over two hour periods at night than during the day (mean km/2 h ± S.E.: AA day = 0.71 ± 0.06, AA night = 2.63 ± 0.10, MS day = 0.67 ± 0.02, MS night = 1.70 ± 0.07 (Fig. 1E,F, S1); linear mixed model for ‘Day vs Night’: t = 21.85, p < 0.001 (Table 1)). Birds in the AA ecosystem moved greater distances than those in the MS ecosystem (linear mixed model for ‘Ecosystem: AA vs MS’, t = 2.40, p = 0.02 (Table 1)). In both ecosystems, birds flew largely continuously throughout the night (Fig. 1E,F), and almost all long distance flights (>50 km in 2 hours, n = 25) occurred at night; the exceptions were one instance during the day by one individual in the AA ecosystem and one instance in the MS ecosystem (Fig. 1E,F). On average, birds flew a greater distance at night than during the day over the time they were tracked (Fig. 1E,H). Comparing a subset of the data (5 months with 5 individuals continuously present) the difference between night and day cumulative flight distances was not significant in the MS ecosystem (two sample t-test: t = −1.22, p = 0.27, day mean: 336.91 km, night mean: 490.58 km) but was significant in the AA ecosystem (two sample t-test: t = −4.49, p < 0.01, day mean: 243.77 km, night mean: 838.64 km). Maximum flight displacements of around 100 km in two hours indicated that flight speeds of up to 50 km/h or more were possible (Fig. 1E,F).

**Table 1:** **Top rows:** Output of the linear mixed effect model looking at the displacement by Pacific black duck in both ecosystems (AA: arid aseasonal, MS: mesic seasonal) during day and night (movements <0.1 km were excluded; n = 9.8 k for day and n = 8.4 k for night). Bottom rows: Output of the linear mixed effect model looking at the Nocturnality Index in response to large rainfall events in both ecosystems.

Fixed Effects	Estimate (S.E.)	t	p
Day vs night	1.383 (0.06)	21.85	<0.001
Ecosystem: AA vs MS	0.673 (0.28)	2.40	0.02
Post Large Rainfall Period	−0.021 (0.03)	−0.63	0.53
Ecosystem: AA vs MS	0.002 (0.03)	0.07	0.94
Ecosystem x Post Large Rainfall Period	0.188 (0.04)	4.71	<0.001

### Effects of rainfall on flight distances

**Figure 2:**
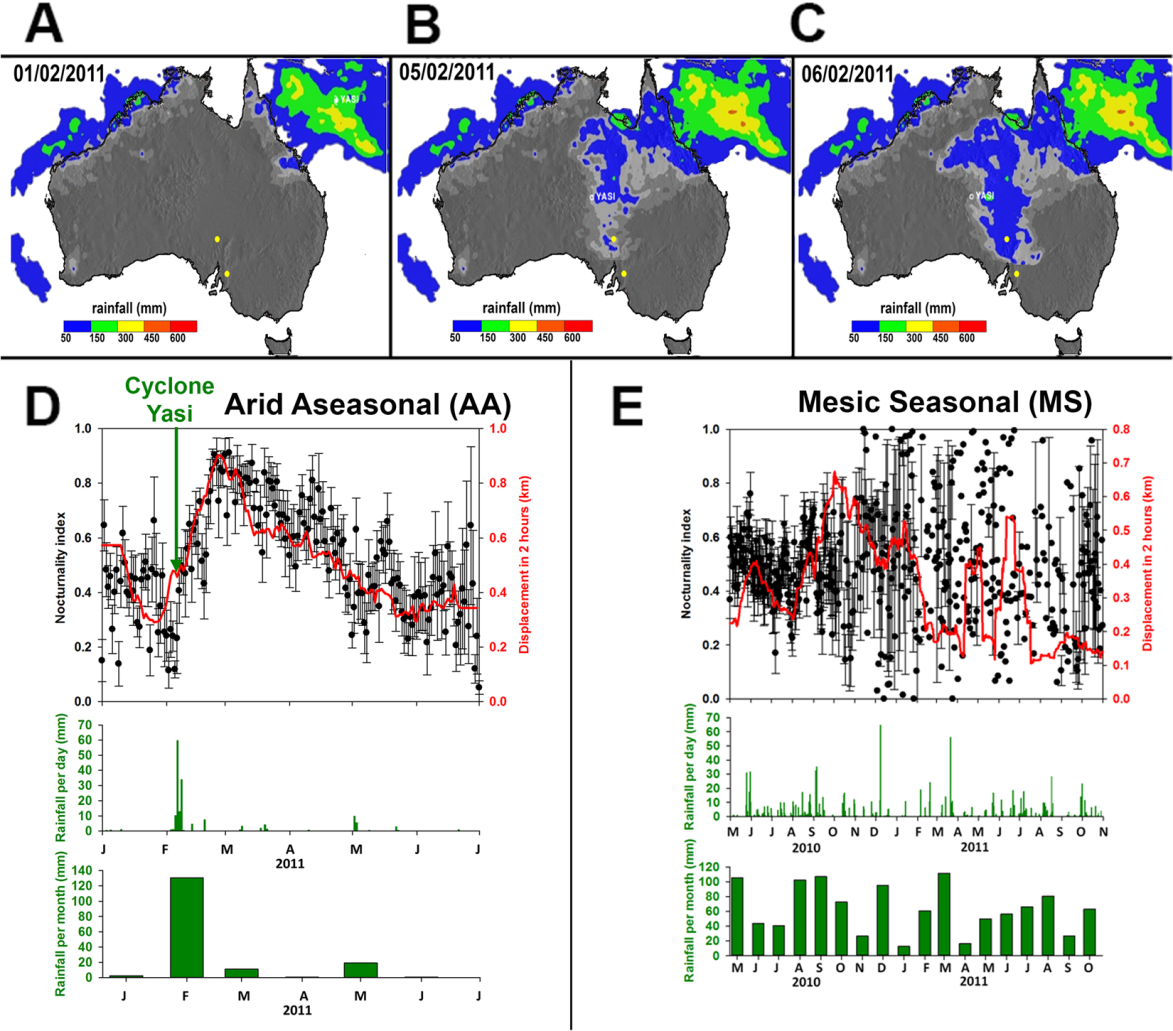
Panels A-C are rainfall maps showing cyclone Yasi’s movement across Australia, 1–6 February 2011, and onto our study sites. Colours indicate rainfall (mm) during 4 hours. Images from ©NASA (http://trmm.gsfc.nasa.gov/trmm_rain/Events/australia_tc_rain_jan-apr_2011_movie.gif). Panel D is for the AA ecosystem, and shows movement indicated by displacement during two hours (shown in red), daily nocturnality index (S.E.) (shown in black), and daily rainfall (shown in green), all plotted against month during 2011 (January – August) including the arrival of cyclone Yasi (n = 22.2 k GPS fixes); beneath monthly rainfall. Displacement was smoothed using a running average with sampling proportion of 0.1 in Sigmaplot 12.0. Rainfall was the mean daily rainfall from three nearest stations. Panel E is as for panel D but for the MS ecosystem and n = 31.7 k GPS fixes; daily rainfall mean is from four nearest weather stations.

Between 6 and 8 February 2011, the powerful cyclone Yasi, which occurred during a strong La Niña period of ENSO, produced a large rainfall event in the AA ecosystem (Fig. 2A–D), with > 100 mm of rainfall over three days. In the MS ecosystem during the period of our study, there were two large rainfall events comparable in size (daily rainfall of 65 mm on 8 December 2010, and 56 mm on 21 March 2011, Fig. 2E). Yet, despite these comparable rainfall events, birds did not respond in a similar way to those in the AA ecosystem. In the AA ecosystem, the total distance flown during a 24 hour period increased after the large rainfall event, with steep increases in displacement in two hours (Fig. 2D), but in the MS ecosystem this did not happen (Fig. 2E). Similarly, the proportion of flight that was during the night also increased markedly and significantly after rainfall in the AA ecosystem, as indicated by the steep rise in the Nocturnality Index (NI) (Fig. 2D), which was a proportional measure of flight activity at night versus day: NI reached almost 90% in the first days after the large rainfall event (Fig. 2D). There was an increase in NI during the 28 days after rain, but only in the AA ecosystem (Table 1), with an average increase of 19% ± 0.03 (S.E.); there was no such increase in the MS ecosystem (Fig. 2D,E, Table 1). The differential effect of ecosystem on NI is supported by linear models showing that the predictor variables (‘Post Large Rainfall Period’ and ‘Ecosystem’) were only significant when in interaction with each other (estimate = 0.188 ± 0.04 (S.E.), t = 4.71, p < 0.001; Table 1). After cyclone Yasi, in the AA ecosystem there was also strong divergence in the cumulative distances flown at night versus during the day, consistent with a facultative increase in night-time flight (Fig. 1G). NI and the mean displacement in two hours dropped off slowly in the three months following cyclone Yasi (Fig. 2D), as wetland habitat dried up.

### Nocturnal flight locations

Birds in the AA ecosystem visited a greater number of locations at night than during the day (Negative binomial GLMM: ‘Day vs Night’: df = 23, z = 4.230, p < 0.001 (Tables S1 and 2)). The number of locations visited increased after the large rainfall event (cyclone Yasi) (‘After vs Before cyclone Yasi’: df = 23, z = 1.967, p = 0.04; (Table S2)). The increase was especially pronounced at night, with a 4.5 fold increase (Table S1) although this interaction was marginally non-significant in the linear model (‘Day vs Night’ x ‘Before and After cyclone Yasi’: df = 1, 24, z = 1.765, p = 0.077). In the 28 days before cyclone Yasi, birds moved within a small portion of their local area (Figure S2, top left panel), which was centred almost entirely on permanent water at the trapping site. In the 28 days after cyclone Yasi, there was increased use of surrounding areas (Figure S2, top right panel); birds visited more locations, primarily at night, and over a larger area than before cyclone Yasi. The top two panels show intense activity around the trapping site; there was a large (58%) increase in area of the utilisation distribution after the large rainfall event. Utilisation distributions (Figure S2, top two panel) for the before and after periods overlap by 47%. An example of one representative individual’s trajectory before and after cyclone Yasi is presented (Figure S2, bottom two panels).

**Figure 3:**
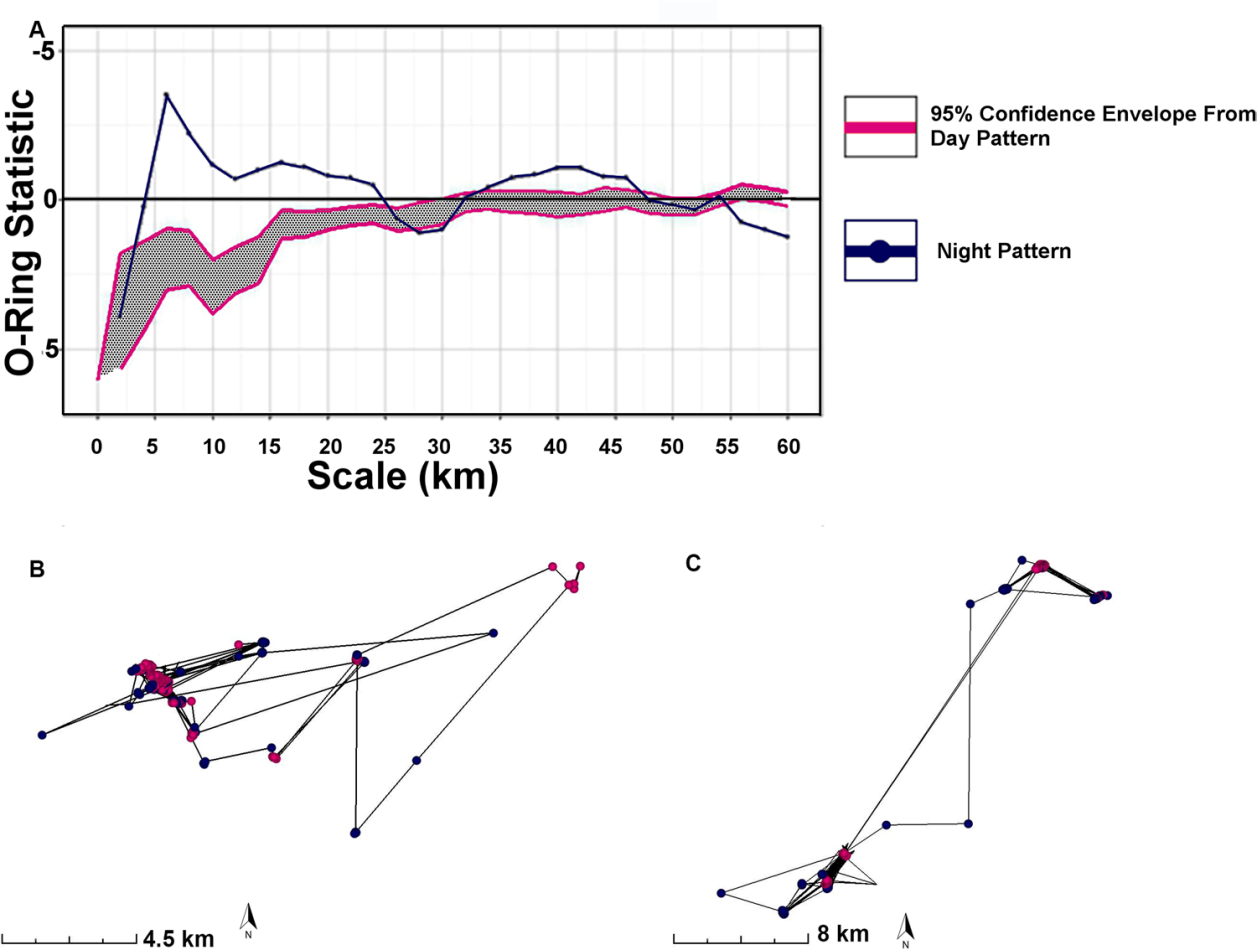
Data revealing how distribution of locations visited by Pacific black duck during daytime and night differ markedly. All data here from AA ecosystem. Panel A shows the O-ring statistic plotted against distance from the release point, and so describes the average density of GPS fixes at a distance^70^. As indicated by divergence above the shaded confidence envelope, the night-time point pattern is more dispersed than the daytime one; this occurs both at 2–24 km and 32–48 km (n = 23.2 k GPS fixes). Panels B and C each show data from an individual Pacific black duck revealing, in accordance with panel A, that daytime locations (pink) are less dispersed than night-time locations (blue).

Generally, at night birds in the AA ecosystem visited small and medium sized local water bodies within a 60 km radius of the point of release (Fig. 3A–C). Point pattern analysis of the clustering in GPS fixes revealed that while day-time locations were tightly clustered around centres of activity, nocturnal locations were more widespread with larger clusters and a smaller number of centres of activity (Fig. 3A–C; Table S3). Locations visited at night were more dispersed, as shown by lack of overlap of the confidence envelope of day with night (Fig. 3A); this lack of overlap occurred both on fine spatial scales (2–24 km radius from release point; Fig. 3A) and at coarser scales (32–48 km scale; Fig. 3A). Examples from two representative individuals are presented (Fig. 3B,C).

## Discussion

Our study is the first to show that waterfowl can facultatively adjust levels of nocturnal flight in response to intense rainfall, and we show the effect varied with ecosystem. Birds in an arid aseasonal ecosystem showed a marked increase in the amount of nocturnal flight, the total distance flown daily, and the number of sites visited at night following a major rainfall event. In contrast, birds in the mesic seasonal ecosystem showed no such response, despite similar sized rainfall events. Other key findings emerging from our study were that: 1) ducks flew during all hours of the night, not just around dawn and dusk as predicted from earlier studies^44–46^; 2) long-distance flights occurred almost exclusively at night and the cumulative distance travelled at night was higher than during the day; and 3) ducks visited more locations at night, different locations and ones that were more dispersed than those visited during the day, at least in the arid aseasonal ecosystem.

Studies on captive migratory passerines have shown increased nocturnal flight and activity during migration^14,15,47^, but attempts to quantify nocturnal behaviour in free living wild birds have been largely limited to activity budgets and counts of foraging individuals^48–50^. Our GPS satellite tracking and determination of displacement and sites visited on an hour-by-hour basis illustrates an approach that can provide important new insights into nocturnal flight and movement ecology of birds, and our findings complement a recent study of this species^51^.

Owen^50^ reported nocturnal feeding in a range of northern hemisphere waterfowl species including some members of the genus *Anas*. Since then evidence has emerged that waterfowl, from the northern hemisphere at least, switch habitat around dawn and dusk^44–46^ and perform nocturnal foraging^52,53^. However, the hypothesis that nocturnal movement in waterfowl represents a simple habitat switch, namely a point-to-point back and forth ‘commute’ between specific sites, has been tested in few species, and often without detailed movement data^54^. Our study revealed that Pacific black ducks flew at night around their local environs extensively and throughout the night, not just during dusk and dawn as expected from earlier work^20,21,44–46^. The high temporal sampling rate and accuracy of our GPS data allowed point pattern analysis (Fig. 3), that to date has rarely been applied to movement data^41,55,56^.

Why do Pacific black duck fly during the night? Modelling and work on other species suggests three main functional hypotheses which are not mutually exclusive. One is that waterfowl and other species gain predator protection particularly from diurnal raptors^53,57^. A second, especially plausible in arid hot ecosystems as in our study, is that nocturnal flight aids heat dissipation, and reduces heat stress and water loss^58^. A third is that nocturnal flight facilitates accurate navigation as there are multiple reliable navigational cues, such as a stellar compass in addition to geomagnetic and visual cues^59^. Tests of these hypotheses on Pacific black duck have yet to be conducted. As to the function of increased nocturnal flight in the arid aseasonal ecosystem after heavy rainfall, the most likely hypothesis is an improved ability to find and exploit the time-limited rich resources for feeding and breeding created by such rainfall. In birds, levels of nocturnal activity are usually controlled by a combination of internal (physiological) and external (environmental, meteorological, ecological) factors interacting to influence flight and movement^60^. Further work to elucidate the control of facultative nocturnal flight in this, and related species, is needed.

Intense rainfall in the arid inland of Australia, such as with cyclone Yasi, can create large areas of breeding and feeding habitat for waterfowl^33^. Pacific black duck responded by increasing the daily distance they flew and the proportion of this flight distance that was nocturnal. These elevated levels persisted for weeks and then slowly declined to baseline levels. At night, more locations were visited than during the day, and birds visited the newly created habitat throughout the night, rather than during the day, when they remained relatively sedentary. While animals can adjust behaviours in response to landscape level changes in resource distribution^37,41,61^, our study showed that a marked increase in nocturnality occurred in arid aseasonal ecosystems after a rapid change in environment (rainfall). By contrast, Pacific black duck in the mesic seasonal ecosystem did not show facultative nocturnal flight despite similar amounts of rainfall. Whether the difference in flight behaviour of birds in the two ecosystems is triggered by the different abiotic factors of the environment, or whether there are population differences, or some combination, remains unknown. Pacific black duck (like many Australian waterfowl species) do not have a clearly defined breeding season and can breed at almost any time of year if conditions are favourable, particularly in the arid interior. In this study we have no direct evidence of breeding by tagged individuals and have no information on their life history stage beyond the fact that all individuals followed were adults. While a search for profitable feeding patches in an arid environment is an obvious driver of such movement, it is probable that extensive nocturnal flights may have been driven, to some extent, by the search for habitat with sufficient food resources to undertake a breeding attempt.

Analyses of locations visited by Pacific black duck in the arid aseasonal ecosystem revealed that, while daytime activities were tightly clustered around a small number of water bodies, nocturnal movements were more loosely clustered. This might be explained by a small number of permanent water bodies acting as a daytime roost for most waterfowl in the area, with birds tending to visit a larger number of outlying smaller water bodies at night. Whatever the reason, it is clear that areas and habitat exploited at night can be substantially different to those occupied during the day. Our work therefore supports other studies^53,62^ in concluding that nocturnality in waterfowl needs to be much better understood in order to make effective conservation decisions, such as on the location of protected wetlands.

In Pacific black duck, recent work shows antibodies against avian influenza in up to 57% of birds in inland and south eastern Australia near to our study, with avian influenza prevalence of up to 9% (Klaassen pers. comm.) Avian influenza prevalence and avian influenza antibody prevalence in Pacific black duck, and other anseriformes of inland and south eastern Australia, were found to be linked to rainfall and ENSO^63^. There is global interest in knowing if large scale global climatic phenomena such as ENSO events trigger changes in waterbird movements and if this affects risks of human influenza outbreaks, as has been hypothesized^25,26^. There is also interest in knowing whether and how birds and other animals respond to ENSO events. Consequently, our study may be of interest and relevance to predicting avian influenza risks.

Our findings suggest that nocturnal flight and nocturnal behaviours of waterbirds might be more common than previously thought. Our data also emphasize the potential role of flexibility in nocturnal flight as an adaptive response to rapid changes in the local environment. Facultative control of nocturnal flight in waterfowl may be an adaptation to rapid changes in environmental conditions.

## Methods

### Study areas

The study was conducted in two ecosystems with contrasting ecological conditions matched for longitude (Fig. 1A,B) and as near as possible in latitude (400 km apart). The Arid Aseasonal (AA) ecosystem was centred on the trapping and trapping site of Roxby Downs, South Australia (30.559°S, 136.879°E) and the Mesic Seasonal (MS) ecosystem on Clare, South Australia (33.823°S, 138.607°E). The AA ecosystem has almost the lowest annual mean rainfall found anywhere in Australia (Fig. 1A) and the most extreme variability in rainfall (Fig. 1B). Monthly total rainfall in the AA ecosystem (Fig. 1C)^64,65^ has no seasonal pattern. In the MS ecosystem by contrast, there is high annual rainfall (Fig. 1A) with a strong seasonal pattern, increasing during winter (Fig. 1D). Both AA and MS have seasonal variation in temperatures, which are lower during winter (Fig. 1C,D). All weather data were taken from the Australian Bureau of Meteorology (BOM) records, and for AA and MS ecosystems, from weather stations within 8 km of the trapping sites (1.5 km in the MS ecosystem).

### GPS tagging and data recording

Pacific black ducks (n = 38) were caught at the AA (n = 20) and MS (n = 18) trapping sites and each bird fitted with a harness^66^ attached to a solar powered GPS transmitter (22 g and 30 g, Microwave Telemetry, Columbia, MD, USA, representing about 2.2% and 3% of body weight respectively). GPS fixes at 2 hourly intervals were obtained for up to 20 months (May 2010 to January 2012), and from this the displacement every 2 h of every individual was calculated from the straight-line distance between consecutive GPS fixes (Fig. 1E,F). We used civil twilight (Geoscience Australia, http://www.ga.gov.au/geodesy/astro/sunrise.jsp) to define first light and last light, and if a 2 h period between GPS fixes spanned civil twilight, then the longer duration determined the night/day categorization of that fix. The mean cumulative displacement per individual (Fig. 1G,H) was calculated from adding the mean displacement in 2 h per individual that was still tagged, and separated into day and night periods. For occasional missing GPS fixes, we corrected by using the straight line distance between temporally adjacent GPS fixes from that individual to calculate the mean displacement per 2 h. All analyses (except those indicated below) were performed on GPS fixes within a 60 km radius of the two trapping sites; this allowed us to be confident that all analysed birds were experiencing similar rainfall, day-length and other environmental conditions. Additionally, preliminary data suggested that 60 km was the maximum distance that an individual could travel within a 2 h period and still return to their original location. The criteria yielded a data set of 22.2 k GPS fixes from the AA ecosystem (n = 20 birds) and 31.7 k GPS fixes from the MS ecosystem (n = 18 birds). The exceptions to the above criteria (of only using data from 60 km of the trapping site) were data and analyses summarized in Fig. 1E–H, S1 and Table 1 (top rows) which included data regardless of distance from the trapping site. All displacements of less than 0.1 km were ignored as they most likely represented small non-flying movements within ponds. Data on tagged individuals were available in the AA ecosystem for December 2010 to July 2011 and in the MS ecosystem for May 2010 to January 2012.

### Statistical analyses

Displacement during night and day were compared using a linear mixed effect model with displacement in 2 h as a response, and ‘Night vs Day’ and ‘Ecosystem’ as predictors (Table 1). Individual identity was added to the model as a random effect to control for individual variation in behaviour.

To investigate changes in the amount of day and night flight over time, the Nocturnality Index (NI) was calculated (Fig. 2D,E) based on the following formula

$$NI=\frac{night\,displacement}{night\,displacement+day\,displacement}$$

where ‘night displacement’ equals the vector distance moved per bird during the night, and ‘day displacement’ equals vector distance moved per bird during the day. Nocturnality index of a sample of birds was based on the mean of individual birds means, for any given day.

A linear mixed modelling approach was also used to analyse NI in response to rainfall events, with NI as a continuous response variable. In the AA ecosystem one large rainfall event occurred over the course of three days (>100 mm on 6–8 February 2011, Fig. 3D). In the MS ecosystem two large rainfall events occurred (>50 mm on 8 December 2010 and on 21 March 2011, Fig. 3E). In order to determine the effect of large rainfall events on NI, a 28-day period before and after these large rainfall events was taken to create a binary predictor ‘Post Large Rainfall Period’. In this way, NI during the 28 days before and 28 days after the large rainfall event could be compared; the duration of 28 days whilst arbitrary was considered sufficiently long for any effects of rainfall to become apparent. The model included Post Large Rainfall Period as a binary predictor, ecosystem as a categorical predictor and an interaction term (Ecosystem x Post Large Rainfall Period). Individual identity was entered as a random effect to control for individual variation.

In support of statistical models of movement distances at night and during the day we show cumulative distance moved across time in each ecosystem (Fig. 1G,H). Statistical comparisons of cumulative distance moved in different ecosystems across the entire study period were confounded by different numbers of individuals being present in each location at different stages of the calculation (due to different deployment dates and survival rates). We compared the difference between cumulative distance moved across a sub-sample of the data using a paired sample t-test comparing 5 months of data with 5 individuals present in a 60 km area in each ecosystem throughout (AA ecosystem: Jan-Jun 2011, MS ecosystem: Jun-Nov 2010).

A generalized linear mixed model (GLMM assuming a negative binomial distribution with log-link, individual included as a random effect) was used to estimate whether the number of locations visited by individuals in the AA ecosystem differed between ‘Day and Night’ and in a 28-day period ‘Before and After cyclone Yasi’ and if there was an interaction between these two factors. We used a negative binomial distribution and an individual level random effect to account for overdispersion in the data (tested using ‘overdisp_fun’ in R package “lme4”^67^, overdispersion ratio: 1.25, p = 0.18). Utilisation distributions^68^ for each 28-day period, and individual’s trajectories were created via kernel density estimation in the “adehabitatHR” package in R^69^ (Figure S2). The O-ring statistic describes the average density of points at a given distance from each other^70^ and was used to compare the position of night and day GPS fixes in the AA ecosystem (Fig. 3A). Differences between the day and night distribution were assessed as significant by comparing the observed distribution to the confidence envelope generated by Monte Carlo simulations of the null model. Analyses were done using grid-based estimators in the Programita package^70^.

Statistical modelling was carried out in the R programming environment^71^ using the package “lme4”^67^. All p-values are two-tailed and results were considered significant when p < 0.05. This work was carried out under scientific research permits from the South Australian Department of Water, Environment and Natural Resources (permit U25774-3) and was approved by the DEWNR Wildlife Ethics Committee (permit 43/2009).

### Data Accessibility

Data can be obtained by application to A. Bennett.

## Electronic supplementary material

Supplementary Material
