# Supplementary material for: Heavy rainfall triggers increased nocturnal flight in desert populations of the Pacific black duck (Anas superciliosa)
Source: Sci Rep. 2017 Dec 14;7:17557. doi: 10.1038/s41598-017-17859-0 (PMC5730603; doi:10.1038/s41598-017-17859-0)

Heavy rainfall triggers increased nocturnal flight in desert populations of the Pacific black duck (*Anas superciliosa*)

J. F. McEvoy[1*, 2], R. F. H. Ribot[2], J.C. Wingfield[3] and A. T. D. Bennett[2]

Address:

[1] Smithsonian Conservation Biology Institute, 1500 Remount Road, Front Royal, VA 22630, USA

[2] Centre for Integrative Ecology, Deakin University, Locked Bag 20000, Geelong VIC 3220 Australia

[3] Department of Neurobiology, Physiology and Behaviour, University of California

One Shields Avenue, Davis, California 95616, USA

[*]Corresponding author: mcevoyj@si.edu

Supplementary material

Tables

Table S1. Mean number of locations visited by birds in the AA ecosystem shown separately for night and day, and before and after the large rainfall event (cyclone Yasi).

Period	Mean No. Locations (S.E.), Day	Mean No. Locations (S.E.), Night
Before cyclone Yasi	2.4 (0.5)	3.4 (1.3)
After cyclone Yasi	4.9 (0.9)	15.4 (2.9)

Table S2. Output of the negative binomial GLMM for the number of locations visited by birds in the AA ecosystem.

Fixed Effects	d.f.	Z	p
Intercept	3, 24	7.259	<0.001
Day vs Night	1, 24	4.230	<0.001
Before vs After cyclone Yasi	1, 24	1.967	0.04
Day vs Night x Before vs After cyclone Yasi	1, 24	1.765	0.07

Table S3. The O-ring statistic generates a cluster size and number of clusters and is shown for the AA ecosystem, separately for the day and night GPS fixes. Cluster size indicates how dispersed the fixes are around the centres of activity. Number of clusters represents the number of aggregations of fixes nearer to each other than if randomly distributed.

	**Day**	**Night**
Cluster size	0.77	2.18
Number of clusters	20.27	8.73

Figure S1. Displacement in 2 hours (mean ± S.E.) by birds shown separately for each ecosystem (AA and MS) and time of day (day and night); movements <0.1 km were excluded; n = 9,800 for day and n = 8,400 for night.

Figure S2. Top two panels: Utilisation distribution of birds in a 60km radius from the release site in the AA ecosystem, shown separately for the 28 day periods before and after cyclone Yasi's large rainfall event on 6-8 February 2011 (GPS fixes; n = 1500 before and n = 2300bafter). Blue indicates areas of most intense utilisation and red indicates least intense. Bottom two panels: Trajectories for one individual shown before and after cyclone Yasi's large rainfall event; blue indicates night time locations and pink daytime locations.

Figure S1.

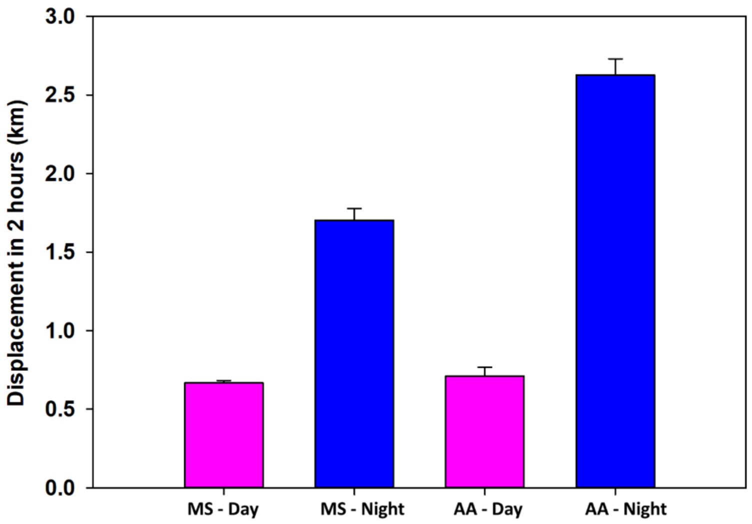

Figure S2.

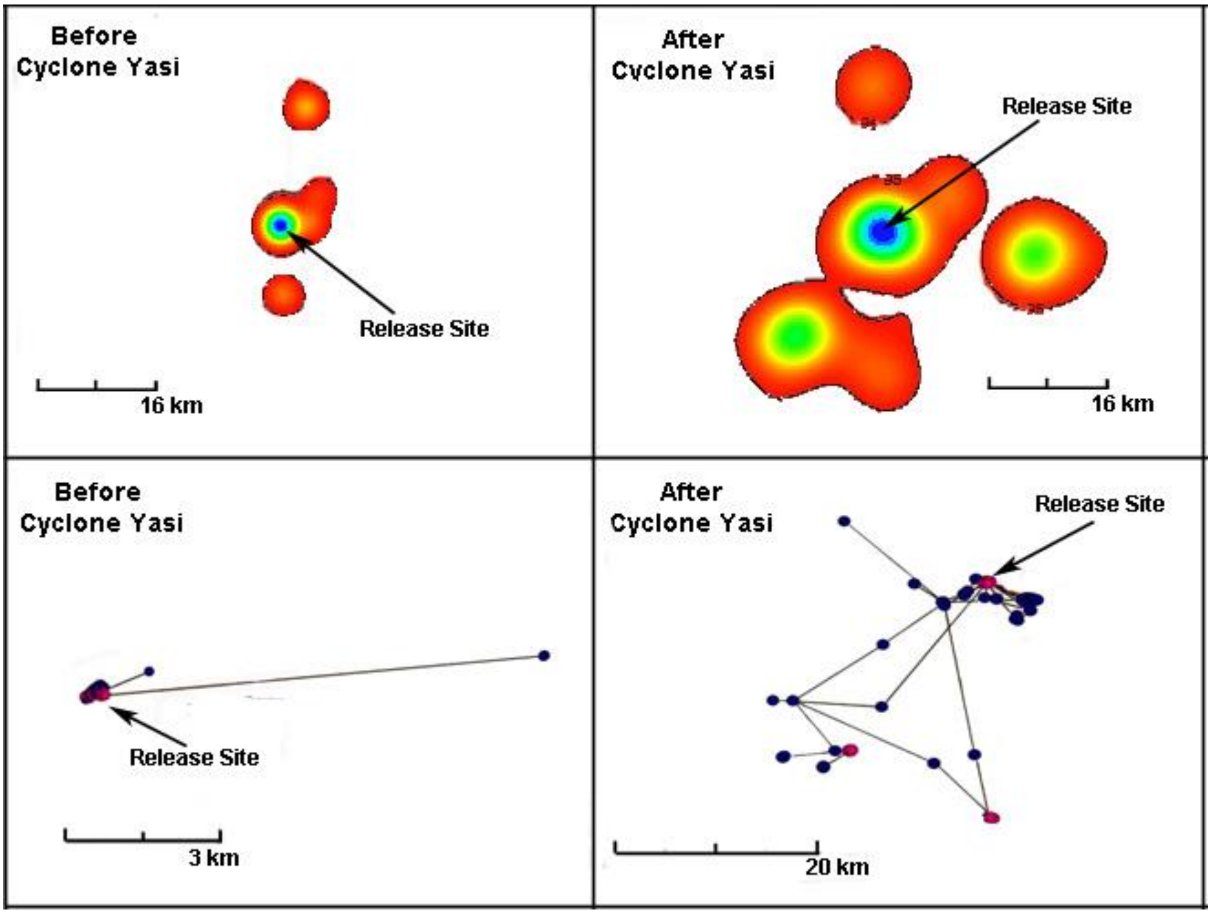

Supplement: Supplementary file 1 — Supplementary Material [file 41598_2017_17859_MOESM1_ESM.pdf]
